# Identification and Characterization of Osmoregulation Related MicroRNAs in Gills of Hybrid Tilapia Under Three Types of Osmotic Stress

**DOI:** 10.3389/fgene.2021.526277

**Published:** 2021-04-06

**Authors:** Huanhuan Su, Jiajia Fan, Dongmei Ma, Huaping Zhu

**Affiliations:** ^1^Key Laboratory of Tropical & Subtropical Fishery Resource Application & Cultivation, Ministry of Agriculture and Rural Affairs, Pearl River Fisheries Research Institute, Chinese Academy of Fishery Sciences, Guangzhou, China; ^2^College of Fisheries and Life Science, Shanghai Ocean University, Shanghai, China

**Keywords:** tilapia, microRNAs, osmoregulation, osmotic stress, expression patterns

## Abstract

Researchers have increasingly suggested that microRNAs (miRNAs) are small non-coding RNAs that post-transcriptionally regulate gene expression and protein translation in organs and respond to abiotic and biotic stressors. To understand the function of miRNAs in osmotic stress regulation of the gills of hybrid tilapia (*Oreochromis mossambicus ♀* × *Oreochromis urolepis hornorum ♂*), high-throughput Illumina deep sequencing technology was used to investigate the expression profiles of miRNAs under salinity stress (S, 25‰), alkalinity stress (A, 4‰) and salinity–alkalinity stress (SA, S: 15‰, A: 4‰) challenges. The results showed that 31, 41, and 27 upregulated and 33, 42, and 40 downregulated miRNAs (*P* < 0.05) were identified in the salt stress, alkali stress, and saline–alkali stress group, respectively, which were compared with those in the control group (C). Fourteen significantly differently expressed miRNAs were selected randomly and then validated by a quantitative polymerase chain reaction. On the basis of Gene Ontology and Kyoto Encyclopedia of Genes and Genomes pathway analysis, genes related to osmoregulation and biosynthesis were enriched in the three types of osmotic stress. In addition, three miRNAs and three predicted target genes were chosen to conduct a quantitative polymerase chain reaction in the hybrid tilapia and its parents during 96-h osmotic stress. Differential expression patterns of miRNAs provided the basis for research data to further investigate the miRNA-modulating networks in osmoregulation of teleost.

## Introduction

MicroRNAs (miRNAs) are a class of 18–25 nucleotide length non-coding small RNAs that are partially or completely complementary to untranslated regions of their target genes. It played regulatory roles by repressing messenger RNA (mRNA) translation or by inducing mRNA degradation post-transcriptionally and involved various biological processes (Huntzinger and Izaurralde, 2011). Because the first miRNA lin-4 and the second let-7 were discovered in *Caenorhabditis elegans* (Wightman et al., 1993; Reinhart et al., 2000), numerous miRNAs have been found in many species in the past two decades (Hafner et al., 2008; Yu et al., 2008; Li et al., 2010). According to the miRBase database, released on March 22, 2018 (miRBase 22)^1^, 38,589 entries representing hairpin precursor miRNAs and 48,885 mature miRNAs have been recorded in 271 species. Numerous researches have shown that miRNAs play dominating roles in multiple biological pathways, such as individual development (Wienholds et al., 2005), abiotic stress responses (Li et al., 2014), pathogen defense, and innate immune responses (Martín-Gómez et al., 2014). Also, Friedman et al. (2009) found that over two-thirds of human protein-coding genes were predicted as target genes of miRNAs.

In the past two decades, with the rapid improvement of genomic and bioinformatics analysis techniques, especially the second-generation sequencing technique, the sequencing of economically and ecologically important animals has been greatly facilitated. Based on RNA-seq analysis, next-generation sequencing has been comprehensively used to reveal the expression patterns of miRNAs and genes under different conditions (Bellin et al., 2009; Ekblom and Galindo, 2010). High-throughput sequencing technologies have also been used to identify conserved and novel miRNAs in aquatic animals, such as *Oryzias latipes* (Qiu et al., 2018), *Gadus morhua* (Bizuayehu et al., 2015), *Oncorhynchus mykiss* (Ma et al., 2012), and *Procambarus clarkia* (Du, 2016). In tilapia, Wang et al. (2016) found 76 differentially expressed miRNAs and 5,559 differentially expressed genes in the ovary compared with those in the testis. Tao et al. (2016) identified 635 mature miRNAs in tilapia gonads at an early stage of sex differentiation. Additionally, several studies have indicated that some aquatic animal miRNAs respond to osmotic stress and that some miRNA targets are osmoregulation-related genes in *Portunus trituberculatus* (Lv et al., 2016) and *Crassostrea* oysters (Zhao X. et al., 2016).

Tilapia, one of the most farmed and studied teleost fish due to its strong adaptability to changing environmental conditions, has been introduced to more than 80 countries (Molnar et al., 2008). One new tilapia strain, named “Guangfu No. 1” (GS-02-002-2015) in China, was a hybrid of *Oreochromis mossambicus* female and *Oreochromis hornorum* male, could grow well in saline pools (Zhu et al., 2018). The 96-h median lethal salinity and alkalinity of the hybrids were 33.74 and 4.96‰ after direct transfer from freshwater to salinity and alkalinity water, respectively (Liu et al., 2015). However, in the chronic salt test, the median lethal salinity was 112.93 ± 4.87‰ by a daily increase in the salinity of 8‰, and the fatal domestication salinity was 128–136‰ (Liu et al., 2015). It was an interesting question of how the tilapia survive in response to environmental stressors involved with the fluctuation of osmotic stress. Zhao Y. et al. (2016) showed that miR-21 has important functions in the regulation of alkalinity tolerance in Nile tilapia. Yan et al. (2012a) demonstrated that miR-30c, a kidney-enriched miRNA, has a crucial function on osmoregulation in Nile tilapia. They had also found that osmotic stress transcription factor 1 (*ostf1*) is the target gene of miR-429, which participates in osmoregulation in response to osmotic stress (Yan et al., 2012b). However, there still remains largely unknown about the expression profiles of miRNAs in tilapia under different osmotic stress, and their roles in osmoregulation remain unclear.

Numerous genes can be regulated by single miRNA and hundreds of miRNAs can regulate one gene, then forming a complex molecular regulatory network between miRNAs and mRNAs (Hossain et al., 2012). It presents a challenge for studying the potential function of miRNAs. RNA-sequencing is a good method to determine the underlying molecular mechanisms of osmoregulation (Li, 2014). Tilapia is an important commercial euryhaline fish species. What is more, tilapia is a good experimental material for osmotic stress regulation research, but the molecular regulation mechanism underlying different osmotic pressure of tilapia is still unexplored. In this study, we detected four miRNA transcriptomes from the gills of the hybrid tilapia under normal conditions group (control group, C), salinity-stressed group (S), alkalinity-stressed group (A), and salinity–alkalinity-stressed group (SA) using the Illumina HiSeq deep sequencing technology. Then, we used both molecular and bioinformatics methods to comprehensively analyze the expression profiles of miRNA, aimed at discovering the possible regulation mechanisms in response to osmotic stress in the gills of hybrid tilapia. These data will help to uncover the complexity of ion regulatory networks and the molecular mechanism of osmoregulation mediated by miRNAs during osmotic stress.

## Materials and Methods

### Sample Collection

The hybrid tilapia was obtained from the Gaoyao Experimental Base of the Pearl River Fisheries Research Institute, Chinese Academy of Fishery Sciences, Guangzhou, China. Before the experiment, all fishes were acclimated to the laboratory environment (28°C) for 2 weeks until they can well feed. The average weight of the experimental fishes was 51.27 ± 3.78 g. Three treatment groups with three replicates of each were set up. According to the previous research results of our research group (Liu et al., 2015), the tilapias were divided into three groups (90 tilapias per group) and acclimated in saline stress (S, 25‰), alkali stress (A, 4‰), and saline–alkali stress (SA, S: 15‰, A: 4‰) at 28°C. The control group was maintained in freshwater. After 24 h of treatment, 18 tilapias were randomly selected in each group, six fishes were mixed into one sample, and three replicates were set for each group. The gills were dissected and immersed into liquid nitrogen for high-throughput sequencing.

The *O. mossambicus* and *O. hornorum* were obtained from the Gaoyao Experimental Base of the Pearl River Fisheries Research Institute, Chinese Academy of Fishery Sciences, Guangzhou, China. The hybrid tilapia and its parents were put in salinity, alkalinity, and salt–alkalinity change water. After 6, 12, 24, 48, 72, and 96 h of three types of osmotic stresses, with three replicates of each time point, five tissues: gill, intestine, liver, kidney, and muscle of three type fishes were dissected and then used to analyze the relative expression levels of miRNAs and their possible target genes by quantitative polymerase chain reaction (qPCR).

### MicroRNA Library Construction and Sequencing

The total RNA used in miRNA library construction was extracted using TRIzol Reagent (Invitrogen). Then, Agilent 2100 Bioanalyzer (Agilent Technologies, Palo Alto, CA, United States), NanoDrop (Thermo Fisher Scientific Inc.), and 1% agarose gel were used to detect the concentration and quality of total RNA of each sample. In strict accordance with the manufacturer’s protocol (NEBNext^®^ Multiplex Small RNA Library Prep Set for Illumina^®^), next-generation sequencing libraries were constructed using 2 μg of total RNA with an RNA integrity number value above 7.5. Polyacrylamide gel electrophoresis was used to purify the total RNA of each sample; the RNA fractions ranging from 18 to 32 nt in size were collected. Then, the collected RNA fractions were ligated with adaptors and used to generate complementary DNA (cDNA) by reverse transcription, which was amplified subsequently by PCR. Lastly, according to the manufacturer’s instruction, the deep sequencing of the four small RNA libraries was performed based on Illumina HiSeq 2000 at the GENEWIZ (Genewiz, Suzhou, China).

### Basic Analysis of Sequencing Results

After using Cutadapt (Martin, 2011) (version 1.9.1) to remove the technical sequences and get clean data, the length distribution of the clean reads and annotation of non-coding RNAs against Rfam and GenBank databases were summarized and analyzed. The clean reads were mapped to the Nile tilapia genome^2^ using Bowtie 2 (V.2.1.0). miRDeep2 (Friedländer et al., 2008) software was used to discover novel miRNAs from deep sequencing data.

### Differential Expression Analysis of MicroRNAs

Using the DESeq (Anders and Huber, 2012) Bioconductor package, a model based on the negative binomial distribution, the differentially expressed miRNAs were detected. Fold expression of miRNAs was expressed in normalized transcripts per million values. After adjusting using the Benjamini and Hochberg’s approach for controlling the false discovery rate, the *P*-values of miRNAs were set to < 0.05 to detect differentially expressed ones.

### Bioinformatics Analysis

To study the potential functions of differentially expressed miRNAs under different osmotic stress, miRanda was used to predict the target genes of the miRNAs (John et al., 2005). Venny online software, the Kyoto Encyclopedia of Genes and Genomes (KEGG) pathway database^3^ (Minoru et al., 2008), and GO-Term Finder (V0.86) were used to detect further the functions of the differentially expressed miRNAs and possible target genes.

### Quantitative Polymerase Chain Reaction of Mature MicroRNAs

Fourteen miRNAs from significantly differentially expressed miRNAs were selected randomly to validate the results of miRNA sequencing by quantitative PCR. The miRNA (< 200 nt) was extracted using a miRcute miRNA Isolation Kit (TianGen, Beijing, China) according to the manufacturer’s protocol. The first-strand cDNA was synthesized using the miRcute miRNA First-Strand cDNA Synthesis Kit (TianGen) according to the operating manual. RNase-free water was used to dilute the concentration of the cDNA before further PCR analysis.

**FIGURE 1:**
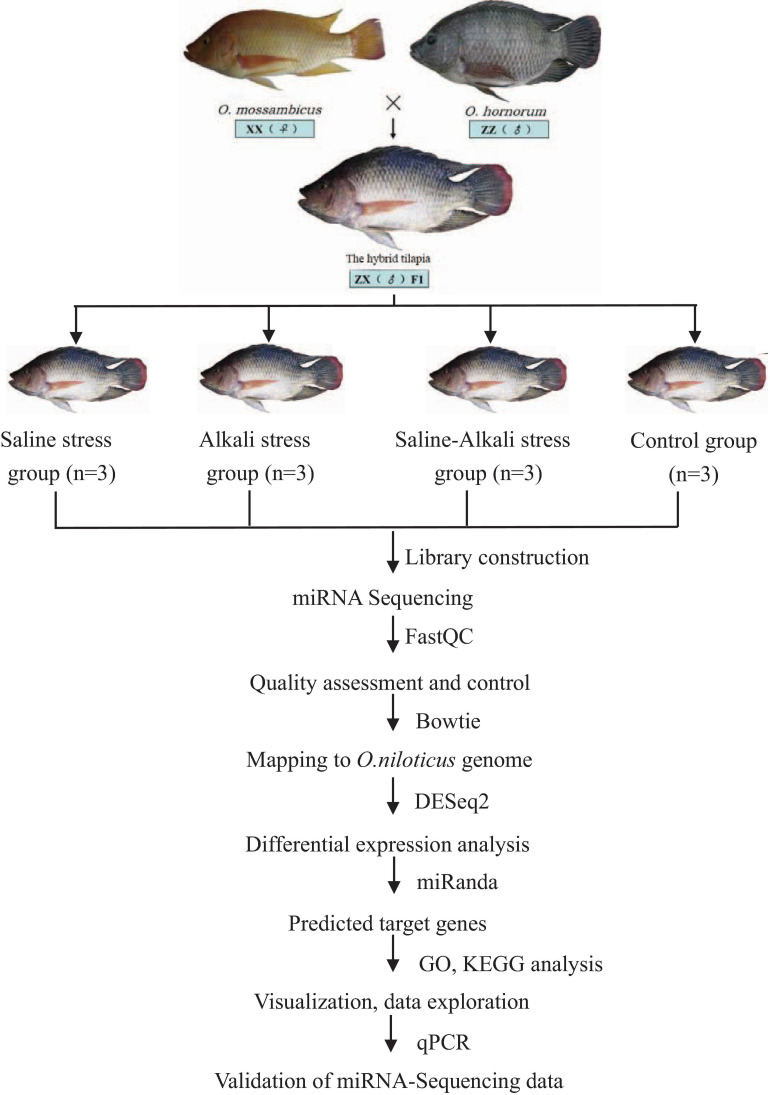
Overview of the experimental design. Flowchart represents miRNA-Seq workflow and bioinformatics analysis workflow.

According to the operating instructions of the miRcute miRNA qPCR Detection Kit (TianGen, Beijing, China), qPCR was performed in an ABI Step One Plus PCR System and in the following conditions: 95°C for 15 min, 40 cycles of two steps (95°C for 5 s, 60°C for 30 s). All primer sequences are listed in Table 1. U6 snRNA was used as the internal control to normalize miRNA expression, and the relative expression level of different miRNAs was calculated using the 2^–ΔΔCt^ method through IBM SPSS Statistics 22. The results are presented as the mean ± standard error. The relative expression levels of miRNAs were analyzed using the one-way analysis of variance method. The workflow of miRNA-Seq and bioinformatics analysis is shown in Figure 1.

**TABLE 1:** Primers of miRNAs used for the qPCR analysis.

miRNAs	Forward primer (5′–3′)
novel miRNA-1052	GGTTCGGCCAGGAAGGGTCTG
novel miRNA-1394	TGGCTCAGTTCAGCAGGAAC
novel miRNA-655	TGCCCTCGAGGACTGGAGTTTG
novel miRNA-531	CGTGATTGCGGATGCTGTGCAGG
novel miRNA-35	TGGAAATCTCTGAATCCCTGCA
novel miRNA-294	TGTACCTCTGCGTGTGTCAGCCG
novel miRNA-306	ATCCCAGCGGTGCCTCCA
novel miRNA-405	TGCCCTCGATAACTGGAGTTTGA
novel miRNA-1053	TGCCCTCGATAACTGGAGTTTGA
novel miRNA-1316	TGCCCTCGAGGACAGGAGTTTG
novel miRNA-1410	TGCCCTCGATAACTGGAGTTTG
novel miRNA-1616	CAGCTGCGGTGCCCGTCA
novel miRNA-1697	TGCCCTCGATAACTGGAGTTTG
novel miRNA-1780	ATGCCCTCGAGGACTGGCGTTT

## Results

### Tilapia Performance Under Saline–Alkali Stress

During the saline and alkali stress, some fishes showed different levels of abnormal behaviors and morphological characteristics. First, some fishes were motionless underwater, and then the tilapia would roll over and be unable to maintain their balance. Also, the body color darkened gradually and secreted a lot of mucus on the body surface. Moreover, the effect of the alkalinity stress and saline–alkali stress on tilapia was more serious than that of salinity stress. Under the alkalinity stress and saline–alkali stress, the head was black, and the gills were bleeding. Only four fishes died in the saline–alkali stress group with swollen abdomen during the experiment. Dissecting the dead fishes observed different degrees of hydrops in the intestines.

### Sequencing and Annotation of MicroRNAs

To identify miRNAs in hybrid tilapia in response to osmotic stress challenges, miRNA libraries derived from four groups were constructed and sequenced. After data quality analysis of the obtained sequence, the total raw reads were obtained from four groups with 12 different libraries, respectively (Table 2). After trimming the adaptor sequences and removing low-quality sequences, all of the clean reads with sizes between 18 and 32 nt were obtained from different groups (Table 2).

**TABLE 2:** Numbers of high-throughput raw reads and clean reads generated from the tilapia gills small RNA library.

Samples	Number of raw reads	Q30 (%)	Number of clean reads	Q30 (%)
C1	56,439,419	99.35	47,979,043	99.32
C5	26,725,751	99.27	24,927,209	99.33
C6	32,598,299	98.81	30,298,937	98.14
S4	19,664,035	97.58	18,063,732	98.2
S5	27,059,933	97.57	24,093,961	98.02
S6	20,009,997	97.23	18,558,648	97.95
A4	162,822,444	99.25	152,382,135	99.19
A5	32,489,499	99.23	31,044,107	99.16
A6	60,981,168	99.25	55,818,260	99.23
SA4	74,516,705	99.21	69,306,040	99.17
SA5	50,394,435	99.23	46,746,342	99.16
SA6	55,634,515	99.25	53,566,296	99.26

Among the 12 libraries, the size distribution and frequency percentage of the clean reads were similar, and 21–23-nt lengths were accounted for approximately 84.2% (Supplementary Figure 1). The 22 nt in length was accounted for 40.13%, which was the most abundant group of the clean reads in the 12 libraries. After mapped to miRbase database, we obtained the largest number of known and novel miRNAs in different groups, which are shown in Table 3. The 11 most abundant known miRNAs in all libraries are shown in Supplementary Figure 2). miR-143-5p, miR-21, miR-203a-5p, miR-203-5p, and miR-22a-5p were the most abundant miRNAs in the control and processed groups.

**TABLE 3:** Number of known and novel miRNAs generated from tilapia gills small RNA library.

Sample	Number of known miRNA	Number of novel miRNA	Sample	Number of known miRNA	Number of novel miRNA
C1	186	379	A4	180	789
C5	182	326	A5	185	391
C6	180	350	A6	186	515
S4	184	254	SA4	187	516
S5	186	296	SA5	186	416
S6	189	270	SA6	190	448

### Significantly Differentially Expressed MicroRNAs Identification Under Osmotic Stress

**FIGURE 2:**
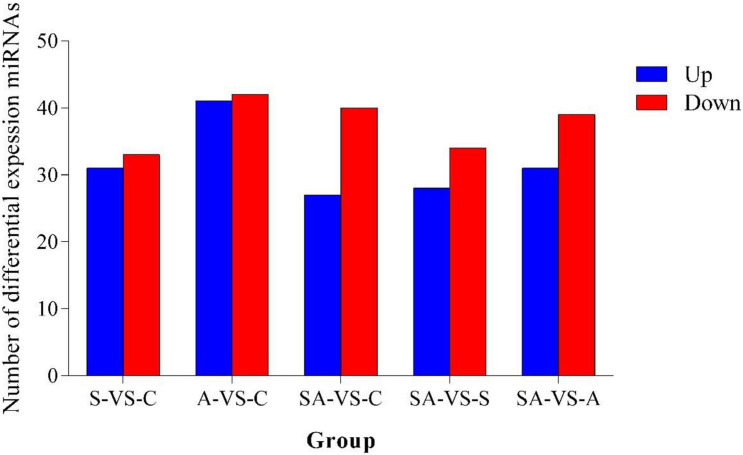
Number of differentially expressed miRNAs in different groups.

**FIGURE 3:**
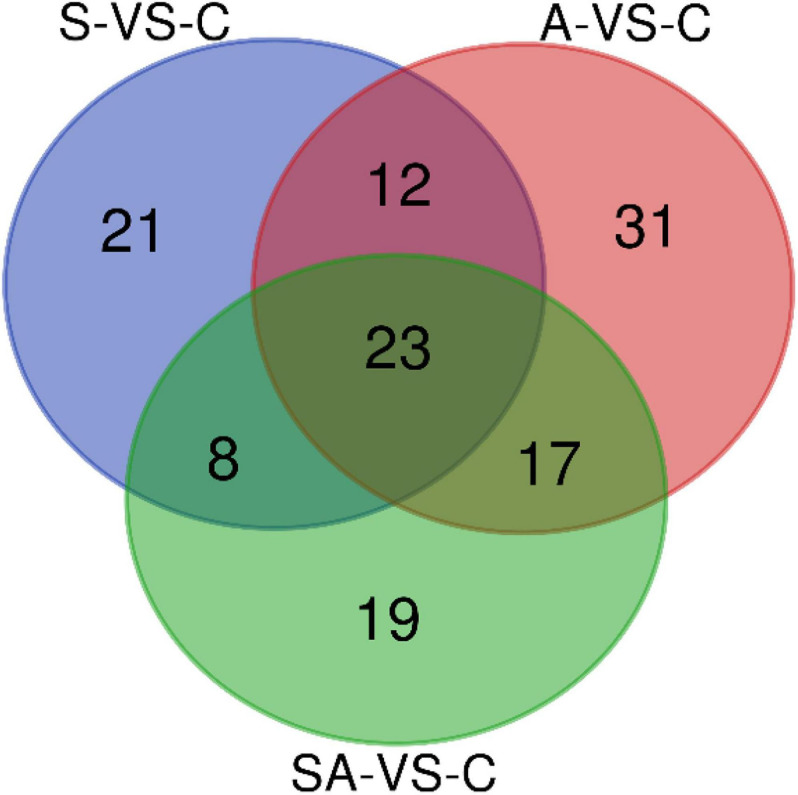
Venn diagram of differentially expressed miRNAs between the different groups.

Based on the criteria that | logFC| > 1 and *P*-value < 0.05, we identified 64 significantly differentially expressed miRNAs in groups C *vs.* S, including 31 upregulated and 33 downregulated miRNAs (Supplementary Figure 3A). Meanwhile, a total of 83, 67, 62, and 70 significantly differentially expressed miRNAs were detected in groups C *vs.* A (Supplementary Figure 3B), C *vs.* SA (Supplementary Figure 3C), S *vs.* SA (Supplementary Figure 3D), and A *vs.* SA (Supplementary Figure 3E), respectively. From those total differentially expressed miRNAs, 42, 40, 34, and 39 were downregulated, and 41, 27, 28, and 31 miRNAs were upregulated, respectively (Figure 2). A total of 23 significantly differentially expressed miRNAs were found in all processed groups through the Venn diagram (Figure 3). In contrast, a total of 12, 8, and 17 miRNAs showed significantly different expression levels in the S & A groups, S & SA groups, and A & SA groups, respectively (Figure 3).

### Identification of Predicted Target Genes

To detect the potential functions of the significantly differentially expressed miRNAs under different osmotic stresses, miRanda was used to predict the target genes of miRNAs. After alignment with the reference genome sequence, a total of 8,061 target genes of significantly differentially expressed miRNAs were predicted in the C *vs.* S group. In contrast, a total of 8,040, 8,771, 8,118, and 6,580 target genes of significantly differentially expressed miRNAs were predicted in groups C *vs.* A, C *vs.* SA, S *vs.* SA, and A *vs.* SA, respectively.

### Gene Ontology Analysis of Predicted Target Genes

The Gene Ontology (GO) database was used to annotate the predicted target genes (Qiang et al., 2017). Among the 8,061 predicted target genes, 1,132 genes participated in GO analysis of group C *vs.* S: 314 (27.7%), 421 (37.2%), and 397 (35.1%) genes were assigned to the cellular components, biological processes, and molecular functioning categories, respectively (Supplementary Figures 4A–E). The results of the GO enrichment analysis (corrected *P* < 0.05) identified 33, 34, and 34 GO terms in the S group, A group, and SA group, respectively. In the salinity group, 64 (20.4%) and 61 (19.4%) genes were enriched in membrane and membrane parts, respectively, within the cellular component ontology, whereas 6 (1.4%) were enriched in response to a stimulus within the biological process ontology. Many genes that may have osmotic regulation functions were enriched in GO terms. Moreover, 32 (8.1%), 192 (48.4%), 3 (0.8%), and 4 (1.0%) were enriched in transporter activity, binding, electron carrier activity, and molecular transducer activity, respectively, within the molecular function ontology.

### Kyoto Encyclopedia of Genes and Genomes Analysis of Predicted Target Genes

By searching the KEGG database, 54 annotated pathways activated in the gills of hybrid tilapia were enriched in group C *vs*. S (Supplementary Figure 5). Meanwhile, a total of 57, 52, 27, and 34 significant pathways were detected in groups C *vs*. A, C *vs*. SA, S *vs*. SA, and A *vs*. SA, respectively. Within these pathways, 20,501 genes were involved in the pathway annotation, within which 1,116 predicted target genes were detected in group C *vs.* S, and 1,273, 1,088, 835, and 814 predicted target genes were detected in groups C *vs.* A, C *vs*. SA, S *vs*. SA, and A *vs.* SA, respectively. At least two pathways were involved in osmotic regulation, including mineral absorption (24 predicted target genes) and the mTOR signaling pathway (26 predicted target genes) in group C *vs.* S (Supplementary Figure 5A). In contrast, in group C *vs.* A (Supplementary Figure 5B), at least three pathways were involved in osmoregulation, including mineral absorption (24 predicted target genes), the prolactin signaling pathway (29 predicted target genes), and the mTOR signaling pathway (33 predicted target genes). Two pathways were associated with osmoregulation, the mTOR signaling pathway (27 predicted target genes) and the prolactin signaling pathway (26 predicted target genes), which were also involved in group C *vs.* SA (Supplementary Figure 5C). Meanwhile, there was one pathway that may be involved in osmotic regulation, the calcium signaling pathway (83 predicted target genes) in group S *vs.* SA (Supplementary Figure 5D). Also, in group A *vs.* SA (Supplementary Figure 5E), ubiquitin-mediated proteolysis (35 predicted target genes) and the calcium signaling pathway (81 predicted target genes) were also found to possibly play important roles in osmoregulation. In Supplementary Figure 5, we only show the top 30 pathways, and if there were not more than 30 terms, all pathways were shown.

Furthermore, we predicted the target genes of differentially expressed miRNAs in each range of the Venn diagram in Figure 3 and conducted GO and KEGG pathway analysis. The pathway analysis suggested that 12 pathways were specifically enriched in the salinity stress, including cell adhesion molecules, regulation of actin cytoskeleton, fatty acid biosynthesis, the calcium signaling pathway, endocrine, and other factor-regulated calcium reabsorption, the citrate cycle (TCA cycle), and so on (Supplementary Table 1). In contrast, in alkalinity stress, there were 15 specifically enriched pathways: ECM–receptor interaction, the cAMP signaling pathway, dopaminergic synapses, the PI3K-Akt signaling pathway, the calcium signaling pathway, the prolactin signaling pathway, the AMPK signaling pathway, etc. (Supplementary Table 1). The adipocytokine signaling pathway, the calcium signaling pathway, the Hedgehog signaling pathway, fatty acid metabolism, the AMPK signaling pathway, the citrate cycle (TCA cycle), etc., were specifically enriched in the mix of salinity and alkalinity challenges (Supplementary Table 1). Also, there were 15, 12, 15, and 22 KEGG pathways that were specifically enriched in S & A, S & SA, A & SA, and S & A & SA, respectively.

### Validation of MicroRNA Sequencing Results by Quantitative Polymerase Chain Reaction

**FIGURE 4:**
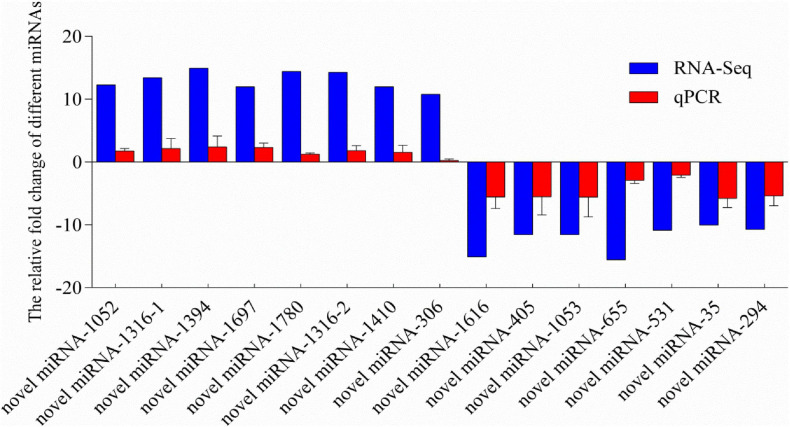
Relative fold change of different miRNAs between qPCR and miRNA-sequencing results. MicroRNA expression levels by qPCR are presented as the fold change compared with the control after normalization against U6snRNA. Relative expression levels from the RNA-seq results were calculated as logFC values. Novel miRNA-1316 had significantly different expressions in two different groups, so we calculated the levels in two groups. (Note: the novel miRNA-1316-1 was in group C *vs.* S; the novel miRNA-1316-2 was in group C *vs.* SA).

To validate the reliability and validity of the miRNA sequencing, 14 miRNAs were randomly selected from the significantly differentially expressed miRNAs for qPCR validation. Melting-curve analysis revealed that primers of all tested miRNAs were specific. The results of qPCR were compared with the miRNA-Seq analysis results (Figure 4). The results of qPCR were significantly correlated with the RNA-sequencing results with a correlation coefficient of 0.94 (*P* < 0.01), indicating the credibility of the miRNA sequencing results.

### Relative Expression Levels of MicroRNAs and Targets in the Hybrid Tilapia and Its Parents

**FIGURE 5:**
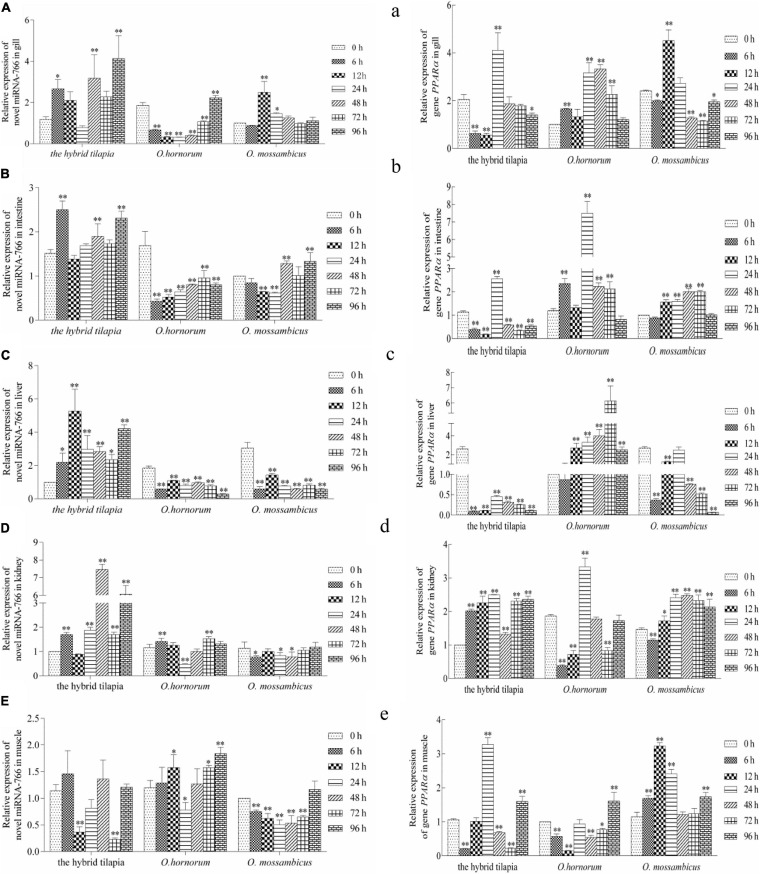
Expression levels of selected novel miRNA-766 **(A–E)** and its target gene *PPAR*α **(a–e)** in the control group (C group) and alkalinity stressed group (A group) in the hybrid tilapia and its parent samples for 96 h. “^∗^” and “^∗∗^” indicate significant differences (*P* < 0.05 and *P* < 0.01, respectively) between values obtained pre-(0 h) and post-alkali stress (6, 12, 24, 48, 72, and 96 h), by paired-samples *t*-test.

**FIGURE 6:**
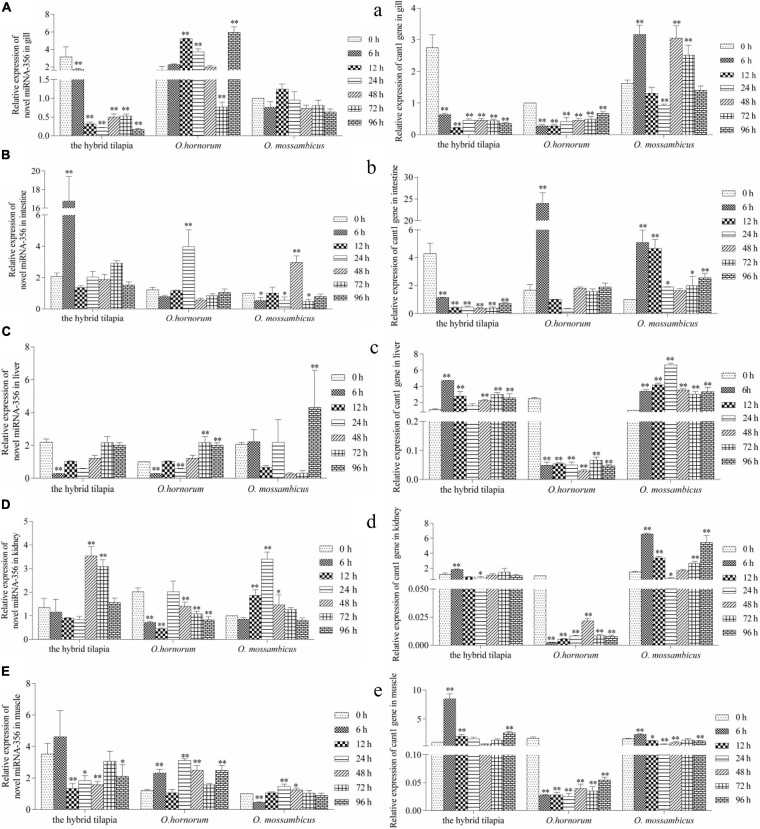
Expression levels of selected novel miRNA-356 **(A–E)** and its target gene *cant1* **(a–e)** in the control group (C group) and alkalinity stressed group (A group) in the hybrid tilapia and its parent samples for 96 h. “^∗^” and “^∗∗^” indicate significant differences (*P* < 0.05 and *P* < 0.01, respectively) between values obtained pre-(0 h) and post-alkali stress (6, 12, 24, 48, 72, and 96 h), by paired-samples *t*-test.

**FIGURE 7:**
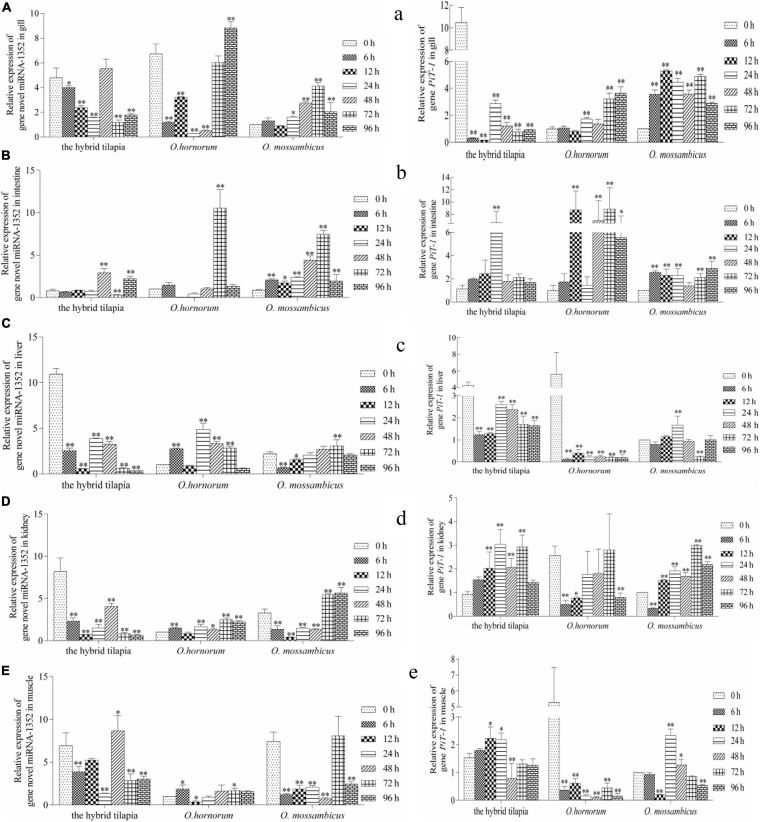
Expression levels of selected novel miRNA-1352 **(A–E)** and its target gene *PiT-1* **(a–e)** in the control group (C group) and alkalinity stressed group (A group) in the hybrid tilapia and its parent samples for 96 h. “^∗^” and “^∗∗^” indicate significant differences (*P* < 0.05 and *P* < 0.01, respectively) between values obtained pre-(0 h) and post-alkali stress (6, 12, 24, 48, 72, and 96 h), by paired-samples *t*-test.

The relative expression levels of miRNAs and its targets: novel miRNA-356–*cant1* (short for “calcium-activated nucleotidase 1”), novel miRNA-766–LOC100709869 (“peroxisome proliferator-activated receptor-alpha,” in short *PPAR*α), and novel miRNA-1352–LOC100694683 (“sodium-dependent phosphate transporter 1-A,” in short PiT-1) were detected by qPCR. The novel miR-766 and novel miR-1352 expression levels first decreased and then increased during 96 h of alkali stress (Figures 5–7). In contrast, the predicted target gene *PPAR*α and PiT-1 expression levels first increased and then decreased. When compared with its parents, the expression levels of novel miR-766 in the hybrid tilapia were higher than those of its parents, but the expression levels of gene *PPAR*α were lower than their parents. Through detected relative expression levels of miRNA and its target genes in gills, intestines, livers, kidneys, and muscles of the hybrid tilapia and its parent, we find that the hybrid tilapia has a better regulation mechanism of saline–alkali tolerance.

## Discussion

Because the roles of miRNAs in biological pathway regulation were unraveled, their roles have been comprehensively researched in many species. However, there are barely reports of miRNA research in hybrid tilapia under salinity stress, alkalinity stress, and salinity–alkalinity stress. In this study, the results of miRNA length distribution found that the 21–23-nt length accounted for proximal 84.2% of the total miRNAs in the control and processed libraries. The length distribution of small RNA sequencing data (Supplementary Figure 1) in the 12 libraries was in coincidence with the results in other aquatic organisms (Lv et al., 2016; Tao et al., 2016; Xu et al., 2016; Zhao X. et al., 2016), suggesting that the results of miRNA sequencing had high accuracy and could be used for subsequent experiments in this study.

We identified many conserved and novel miRNAs (Table 3) in the gills of hybrid tilapia, indicating that each species have their own sets of unique miRNAs and conserved miRNAs (Zhou et al., 2013). The miRNA-143, miRNA-21, miRNA-203, and miRNA-22 families were the most abundant conservative miRNAs in both the control and osmotically stressed groups. These miRNAs had a long evolutionary history and were a widespread and conserved expression in various species, manifesting that they might play crucial roles in essential biological processes in various organisms (Cordes et al., 2009; Huang et al., 2014; Lone et al., 2018; Masoudi et al., 2018). Our results found that miR-146a was upregulated in three types of osmotic stress, and the miR-737 was downregulated after alkalinity and salinity–alkalinity stresses. Taganov et al. (2006) proposed that miR-146 has a key role in the regulation of the Toll-like receptor and cytokine signaling through a negative feedback regulation loop. Numerous studies have shown that the expression levels of miR-146a and miR-146b have also been related to the metastatic, developing, and proliferative responses of various cancer cells, such as papillary thyroid carcinoma (Jazdzewski et al., 2008) and cervical cancer (Wang et al., 2008). Perry et al. (2009) demonstrated that interleukin-1b induced the transcription of miR-146a and miR-146b through different intracellular pathways. Muhammad (2012) found that miR-737-predicted target genes were involved in immune-related transcription factors and metabolism. In this study, the upregulation of miR-146a and downregulation of miR-737 may be implicated with immune suppressive and cell inflammatory responses in the hybrid tilapia gill cells after osmotic stress.

The comprehensive expression analysis of miRNA and its target genes provides a research basis for identifying the regulated functions of miRNA and its target genes under external stimulations. KEGG pathway database was used to investigate further the biological functions and network regulation mechanism of predicted genes. There were 12, 14, 16, 16, 12, 14, and 22 pathways identified in comparison between different groups, and the most frequent pathways were involved in signal transduction and the metabolic system (Supplementary Table 1). Among them, some pathways including ECM–receptor interaction in *Crassostrea oysters* (Zhao X. et al., 2016), dopaminergic synapse in *Scatophagus argus* (Su et al., 2016), MAPK signaling pathway in genus *Mytilus* (Lockwood and Somero, 2011), calcium signaling pathway in *Ruditapes philippinarum* (Nie et al., 2017), cell adhesion molecules in *Crassostrea gigas* (cell adhesion molecules) (Zhao et al., 2012), cAMP signaling pathway in *Callinectes sapidus* (Lohrmann and Kamemoto, 1987), tight junction, fatty acid biosynthesis, and thyroid hormone signaling pathway in *Acipenser baeri* (Guo et al., 2018) have been proven to be related to osmoregulation. GO annotation results of possible target genes demonstrated no significant difference in the regulated function of miRNAs of the target genes under three osmotic challenges.

From the analyzed results of significantly different expression transcripts between the control and processed groups, we selected three functional miRNA–mRNA pairs to detect the possible regulated functions under alkali stress. The relative expression level of miRNAs and predicted target genes in *O. mossambicus*, *O. hornorum*, and the hybrid tilapia were detected through qPCR. The integrated analysis of the expression levels of miRNAs and their predicted targets revealed that the expression trends of miRNAs had negative correlations with their target genes, and the results were coincident with the observations that miRNAs repressing regulates target gene expression through transcript cleavage or translation repression (Guo et al., 2010). By comparing the expression levels of three miRNA–mRNA pairs in gills, intestines, livers, kidneys, and muscles at differently stressed times between the hybrid tilapia and its parents, we found that the hybrid tilapias could rapidly mobilize the expression of miRNAs and their target genes, so we proposed that the hybrid tilapia may maintain good osmoregulation mechanisms during osmotic stress.

Fish can regulate membrane permeability by altering the levels of long-chain polyunsaturated fatty acids (LC-PUFAs) in cell membrane phospholipids to maintain intracellular osmotic pressure balance (Yehuda et al., 2002). In recent years, a large number of studies have shown that environmental salinity can affect the composition of fatty acids in fish tissues, such as *Salmo salar* (Zheng et al., 2005), *Siganus canaliculatus* (Li et al., 2008), and *Pagrus major* (Sarker et al., 2011); the LC-PUFA content under low-salt farming conditions was higher than that of the control group. This fully demonstrates that fish can regulate the synthesis of LC-PUFA in the body by adjusting the lipid metabolism, which is used to increase the content of LC-PUFA in the cytomembrane, changing the ion permeability of the cell membrane to adapt to the salinity of the culture environment. *PPAR*α is activated in the human body by a series of exogenous and endogenous lipids, including various dietary fatty acids (Sanderson et al., 2008), arachidic acid, endocannabinoids, and phospholipids (Chakravarthy et al., 2009). Numerous studies have shown that *PPAR*α is a major regulator of lipid metabolism in the liver (Takashi et al., 2000; Francque et al., 2015; Janssen et al., 2015; Kandel et al., 2016). Chuppa et al. (2018) have shown that *PPAR*α is a target gene of miR-21-5p, which is involved in regulating fatty acid metabolism pathways. Moreover, the decreased expression level of *PPAR*α in the heart associated with inflammation, atherosclerosis, and pathological changes from fatty acid oxidation to glycolysis has been demonstrated in numerous studies (Karbowska et al., 2003; Youssef and Badr, 2004; Guellich et al., 2007). In the early stage of osmotic stress, a large number of ion transporters and enzymes are actively transported (Kang et al., 2012), and in the salinity environment deviating from the isotonic point, 10 to 20% or more of energy is needed to maintain its osmotic pressure balance (Hwang et al., 2011), leading to an accelerated rate of gene synthesis in pathways associated with lipid metabolism. In this study, the expression of the gene *PPAR*α is gradually upregulated, and the expression of genes related to lipid metabolism is activated, changing the permeability of the cell membrane and maintaining the normal osmotic pressure in the alkaline environment.

In this study, we found that the expression levels of sodium-dependent phosphor transporter 1 (*PiT-1*) in the five tissues of the hybrid tilapia and its parents within 96 h of alkaline stress both showed a trend of increasing first and then falling and was opposite to miRNA-1352 expression. This indicates that there may be a targeting relationship between miRNA-1352 and *PiT-1*, which needs further validation. Studies have shown that *PiT-1* is a protein encoded by *SLC20A1* and has functions in phosphate homeostasis, contributes to cellular uptake, cell homeostasis, and mineralization (Atsushi et al., 2011; Lederer and Miyamoto, 2012), and influences the mineralization of human osteoblasts (Barbieri et al., 2018). The knockout of *PiT-1* greatly reduced the matrix mineral deposits in human vascular smooth muscle cells (Dai et al., 2013), indicating that it may play a crucial role in increasing the absorption of minerals by cells and maintaining the stability of cell homeostasis. Furthermore, studies have shown that *PiT-1* can increase Pi-induced apoptosis in vascular mesenchymal cells (Husseini et al., 2013) and is essential for autophagy in vascular smooth muscle cells, both of which are important regulators of vascular calcification. Beck’s research has shown that *PiT-1* silencing can reduce cell proliferation, whereas *PiT-1* mutants overexpressing Pi uptake can rescue this process in HeLa cells (Laurent et al., 2009). In addition, Salaün et al. (2010) showed that *PiT-1* mutants with insufficient Pi uptake could reduce the apoptotic rate of HeLa cells treated with tumor necrosis factor-α. It is indicated that *PiT-1* also plays an important role in the proliferation of fish tumor cells.

*Cant1* is a calcium-activated nucleotidase located in the endoplasmic reticulum/Golgi and hydrolyzes diphosphates and triphosphates in a calcium-dependent manner, which preferentially hydrolyzes UDP to UMP and phosphate (Murphy et al., 2003). Nizon et al. (2012) found that *cant1* localizes to the Golgi apparatus and is involved in glycosaminoglycan synthesis and proteoglycan posttranslational modification. *Cant1* is likely involved in intracellular glycosylation and has been demonstrated for protein quality control and folding in neuroblastoma cell lines (Tito et al., 2012). When *cant1* was knocked down, cell number and DNA synthesis rate were significantly decreased; cell cycle arrest in G1 phase, cell migration rate, and wound healing ability of *cant1* knockdown cells were significantly reduced (Josefine et al., 2011). In this experiment, the expression of the *cant1* gene was downregulated within 96 h of three tilapia stresses and then gradually increased, which may have functions in the proliferation and metastasis of fish cells. The roles of *PiT-1* and *cant1* in the teleost fishes after osmotic stress have not been reported, and we speculated that they play important roles in osmoregulation.

In future studies, we will evaluate the effect on the survival and growth of tilapia under the long-term saline and alkali stress and integrate transcriptome and proteome profiles to understand the mechanism of osmoregulation and the molecular regulatory networks in the hybrids. Furthermore, the tolerance of the hybrid tilapia and its parents under different osmotic stresses may be different in similar developmental stages, so it is important to detect miRNA profiles of the hybrid tilapia and its parents at different osmotic stresses.

## Conclusion

In summary, this study was the first time to analyze miRNA expression profiles of the hybrid tilapia gills under three types of osmotic stress. This research list a vast number of miRNAs and these predicted target genes in the hybrid tilapias under salinity, alkalinity, and salinity–alkalinity stresses. The data presented herein suggest that exposure to osmotic stress decreased immunity and increased the energy consumption in osmotic regulation of the hybrid tilapia. Furthermore, external environmental osmotic stress induced the changes in expression levels of miRNAs and these predicted target genes, which may be involved in osmotic pressure stress regulation signal pathways.

## Data Availability Statement

The datasets generated for this study can be found in the NCBI Sequence Read Archive (SRA) database under SRA accession number PRJNA590373.

## Ethics Statement

The studies involving fish were reviewed and approved by the Animal Care and Use Committee of the Centre for Applied Aquatic Genomics at the Chinese Academy of Fishery Sciences.

## Author Contributions

DM and HS designed the experiment and drafted the manuscript. HS and HZ performed the experiment and analyzed the data. JF participated in experiment and coordination and contributed to the manuscript preparation. All authors reviewed and approved the manuscript.

## Conflict of Interest

The authors declare that the research was conducted in the absence of any commercial or financial relationships that could be construed as a potential conflict of interest.

## Footnotes

1 http://www.mirbase.org/

2 http://asia.ensembl.org/Oreochromis_niloticus/Info/Index

3 http://www.genome.jp/kegg/pathway.html
