# Supplementary material for: Identification and Characterization of Osmoregulation Related MicroRNAs in Gills of Hybrid Tilapia Under Three Types of Osmotic Stress
Source: Front Genet. 2021 Apr 6;12:526277. doi: 10.3389/fgene.2021.526277 (PMC8056028; doi:10.3389/fgene.2021.526277)
Supplement: Supplementary file 1 [file Data_Sheet_1.PDF]

Supplementary Table 1. KEGG pathways associated with osmoregulation in S, A, SA, S & A, S & SA, A & SA, and S & A & SA

Group	Pathway ID	Pathway	Predicted target genes with pathway annotation (579)	P-value
S	ko04514	Cell adhesion molecules (CAMs)	63(10.88%)	3.21E-06
	ko04810	Regulation of actin cytoskeleton	37(6.39%)	7.03E-03
	ko04623	Cytosolic DNA-sensing pathway	11(1.90%)	2.09E-03
	ko04340	Hedgehog signaling pathway	10(1.73%)	7.76E-03
	ko00061	Fatty acid biosynthesis	6(1.04%)	1.67E-03
	ko04721	Synaptic vesicle cycle	54(9.33%)	7.83E-26
	ko04144	Endocytosis	65(11.23%)	1.70E-05
	ko04020	Calcium signaling pathway	56(9.67%)	4.75E-04
	ko04910	Insulin signaling pathway	28(4.84%)	2.70E-03
	ko04961	Endocrine and other factor-regulated calcium reabsorption	13(2.25%)	9.30E-04
	ko00020	Citrate cycle (TCA cycle)	6(1.04%)	1.67E-03
	ko04210	Apoptosis	40(6.91%)	1.13E-07
A	ko04512	ECM-receptor interaction	19(3.58%)	0.0006249
	ko04066	HIF-1 signaling pathway	31(5.84%)	5.51E-06
	ko00260	Glycine, serine and threonine metabolism	7(1.32%)	0.0010052
	ko04024	cAMP signaling pathway	48(9.04%)	0.0084633
	ko04728	Dopaminergic synapse	32(6.03%)	0.0072478
	ko00130	Ubiquinone and other terpenoid-quinone biosynthesis	8(1.51%)	7.69E-08
	ko04151	PI3K-Akt signaling pathway	45(8.47%)	0.0062111
	ko04020	Calcium signaling pathway	47(8.85%)	0.0067489
	ko04668	TNF signaling pathway	18(3.39%)	0.003579
	ko04010	MAPK signaling pathway	53(9.98%)	0.001289
	ko04370	VEGF signaling pathway	15(2.82%)	0.0018646
	ko04917	Prolactin signaling pathway	14(2.64%)	0.0051084
	ko04910	Insulin signaling pathway	47(8.85%)	2.19E-12
	ko04152	AMPK signaling pathway	42(7.91%)	1.07E-11

	ko04120	Ubiquitin mediated proteolysis	30(5.65%)	6.11E-08
SA	ko04810	Regulation of actin cytoskeleton	27(7.07%)	0.0046343
	ko00512	Mucin type O-glycan biosynthesis	7(1.83%)	0.0056598
	ko00270	Cysteine and methionine metabolism	24(6.28%)	4.36E-13
	ko04514	Cell adhesion molecules (CAMs)	66(17.28%)	4.25E-15
	ko04920	Adipocytokine signaling pathway	19(4.97%)	3.31E-06
	ko04020	Calcium signaling pathway	48(12.57%)	1.44E-06
	ko04340	Hedgehog signaling pathway	23(6.02%)	8.12E-15
	ko01212	Fatty acid metabolism	7(1.83%)	0.0041259
	ko04210	Apoptosis	39(10.21%)	1.74E-12
	ko04152	AMPK signaling pathway	17(4.45%)	0.0072756
	ko00020	Citrate cycle (TCA cycle)	4(1.05%)	0.0056776
	ko04961	Endocrine and other factor-regulated calcium reabsorption	9(2.36%)	0.002817
	ko04142	Lysosome	13(3.40%)	0.0068833
	ko04144	Endocytosis	47(12.30%)	2.07E-05
	ko00310	Lysine degradation	26(6.81%)	3.07E-15
	ko04710	Circadian rhythm	14(3.66%)	5.02E-08
S & A	ko00603	Glycosphingolipid biosynthesis - globo and isoglobo series	3(1.08%)	6.89E-03
	ko04922	Glucagon signaling pathway	17(6.12%)	6.72E-03
	ko00020	Citrate cycle (TCA cycle)	4(1.44%)	1.45E-03
	ko04152	AMPK signaling pathway	14(5.04%)	4.14E-03
	ko04144	Endocytosis	49(17.63%)	1.40E-10
	ko00604	Glycosphingolipid biosynthesis - ganglio series	7(2.52%)	5.11E-06
	ko04340	Hedgehog signaling pathway	8(2.88%)	4.58E-04
	ko04210	Apoptosis	38(13.67%)	2.23E-16
	ko04920	Adipocytokine signaling pathway	18(6.47%)	1.07E-07
	ko04910	Insulin signaling pathway	16(5.76%)	2.84E-03
	ko04961	Endocrine and other factor-regulated calcium reabsorption	7(2.52%)	4.18E-03
	ko04020	Calcium signaling pathway	31(11.15%)	6.51E-04
	ko04810	Regulation of actin cytoskeleton	28(10.07%)	1.11E-05
	ko00512	Mucin type O-glycan biosynthesis	6(2.16%)	3.55E-03
	ko03410	Base excision repair	35(12.59%)	6.34E-44

S & SA	ko04514	Cell adhesion molecules (CAMs)	59(13.79%)	1.52E-09
	ko04152	AMPK signaling pathway	21(4.91%)	1.09E-03
	ko04921	Oxytocin signaling pathway	34(7.94%)	2.24E-03
	ko04022	cGMP-PKG signaling pathway	27(6.31%)	8.59E-03
	ko04922	Glucagon signaling pathway	26(6.07%)	1.53E-03
	ko04920	Adipocytokine signaling pathway	26(6.07%)	1.23E-09
	ko01210	2-Oxocarboxylic acid metabolism	5(1.17%)	3.15E-04
	ko04966	Collecting duct acid secretion	8(1.87%)	6.34E-06
	ko04120	Ubiquitin mediated proteolysis	20(4.67%)	1.08E-04
	ko04530	Tight junction	48(11.21%)	2.37E-07
	ko04910	Insulin signaling pathway	23(5.37%)	1.44E-03
	ko04010	MAPK signaling pathway	42(9.81%)	4.72E-03
A & SA	ko04024	cAMP signaling pathway	39(12.26%)	4.74E-05
	ko04961	Endocrine and other factor-regulated calcium reabsorption	7(2.20%)	9.13E-03
	ko00020	Citrate cycle (TCA cycle)	4(1.26%)	2.61E-03
	ko04340	Hedgehog signaling pathway	21(6.60%)	1.61E-14
	ko00061	Fatty acid biosynthesis	9(2.83%)	3.43E-08
	ko04144	Endocytosis	34(10.69%)	2.74E-03
	ko04922	Glucagon signaling pathway	23(7.23%)	2.27E-04
	ko04921	Oxytocin signaling pathway	29(9.12%)	4.88E-04
	ko04920	Adipocytokine signaling pathway	25(7.86%)	8.92E-12
	ko04152	AMPK signaling pathway	24(7.55%)	4.25E-07
	ko04261	Adrenergic signaling in cardiomyocytes	24(7.55%)	7.72E-03
	ko04530	Tight junction	29(9.12%)	1.37E-03
	ko04210	Apoptosis	40(12.58%)	7.63E-16
	ko04330	Notch signaling pathway	17(5.35%)	2.13E-10
	ko04910	Insulin signaling pathway	25(7.86%)	1.79E-06
	ko04330	Notch signaling pathway	11(1.60%)	1.31E-02
	ko04022	cGMP-PKG signaling pathway	66(9.62%)	2.16E-11
	ko04150	mTOR signaling pathway	24(3.50%)	4.47E-03
	ko04725	Cholinergic synapse	43(6.27%)	1.25E-03

S & A & SA	ko04910	Insulin signaling pathway	39(5.69%)	1.85E-05
	ko04152	AMPK signaling pathway	35(5.10%)	2.12E-05
	ko04920	Adipocytokine signaling pathway	37(5.39%)	2.11E-11
	ko00020	Citrate cycle (TCA cycle)	7(1.02%)	9.74E-04
	ko04010	MAPK signaling pathway	83(12.10%)	4.32E-08
	ko05231	Choline metabolism in cancer	25(3.64%)	7.81E-03
	ko04978	Mineral absorption	22(3.21%)	3.42E-10
	ko04062	Chemokine signaling pathway	48(7.00%)	4.39E-05
	ko04917	Prolactin signaling pathway	21(3.06%)	1.34E-04
	ko04370	VEGF signaling pathway	27(3.94%)	1.25E-07
	ko04668	TNF signaling pathway	26(3.79%)	1.25E-04
	ko04014	Ras signaling pathway	45(6.56%)	4.78E-04
	ko04919	Thyroid hormone signaling pathway	30(4.37%)	0.0008294
	ko01521	EGFR tyrosine kinase inhibitor resistance	28(4.08%)	1.01E-06
	ko04514	Cell adhesion molecules (CAMs)	58(8.45%)	5.12E-03
	ko04024	cAMP signaling pathway	99(14.43%)	1.74E-14
	ko04071	Sphingolipid signaling pathway	29(4.23%)	9.84E-05
	ko04620	Toll-like receptor signaling pathway	27(3.94%)	1.17E-05
