# Supplementary material for: Identification and Characterization of Osmoregulation Related MicroRNAs in Gills of Hybrid Tilapia Under Three Types of Osmotic Stress
Source: Front Genet. 2021 Apr 6;12:526277. doi: 10.3389/fgene.2021.526277 (PMC8056028; doi:10.3389/fgene.2021.526277)
Supplement: Supplementary file 2 [file Data_Sheet_2.PDF]

SUPPLEMENTARY FIGURE LEGENDS:

Suppl. FIGURE 1 Length distribution of miRNA sequences in gills of the hybrid tilapia by miRNA sequencing. The most abundant size class was 22 nt, followed by 23 nt and 21 nt.

Suppl. FIGURE 2 Top 11 most abundant miRNAs in the control group (C group) and osmotic-stressed group (S, A, SA group) after treatment 24 h.

Suppl. FIGURE 3 The volcano plot of significantly differential expression miRNAs in gills of hybrid tilapia. The X-axis exhibits differences in fold changes between the two sets of samples (**A**: C vs. S; **B**: C vs. A; **C**: C vs. SA; **D**: S vs. SA; **E**: A vs. SA), and the Y-axis shows the significance of the miRNAs. Red (upregulation) and blue (downregulation) dots represent significantly different expression (P-value < 0.05, $|\log_2^{FC}| > 1$), respectively, and black dots represent no significant differences.

Suppl. FIGURE 4 GO classifications of predicted genes identified from the hybrid tilapia miRNA-Seq data. A: C vs. S; B: C vs. A; C: C vs. SA; D: S vs. SA; E: A vs. SA

Suppl. FIGURE 5 Enriched KEGG signalling pathways of putative target genes identified from the tilapia miRNA-Seq data. A: C vs. S; B: C vs. A; C: C vs. SA; D: S vs. SA; E: A vs. SA

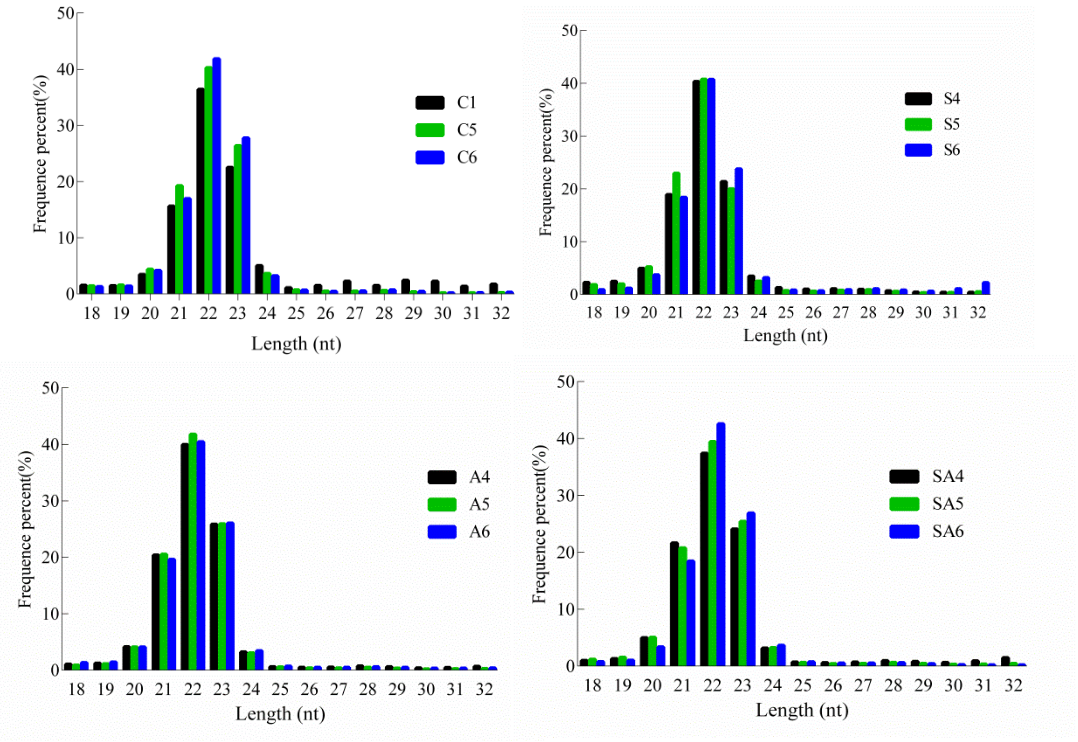

Suppl. FIGURE 1

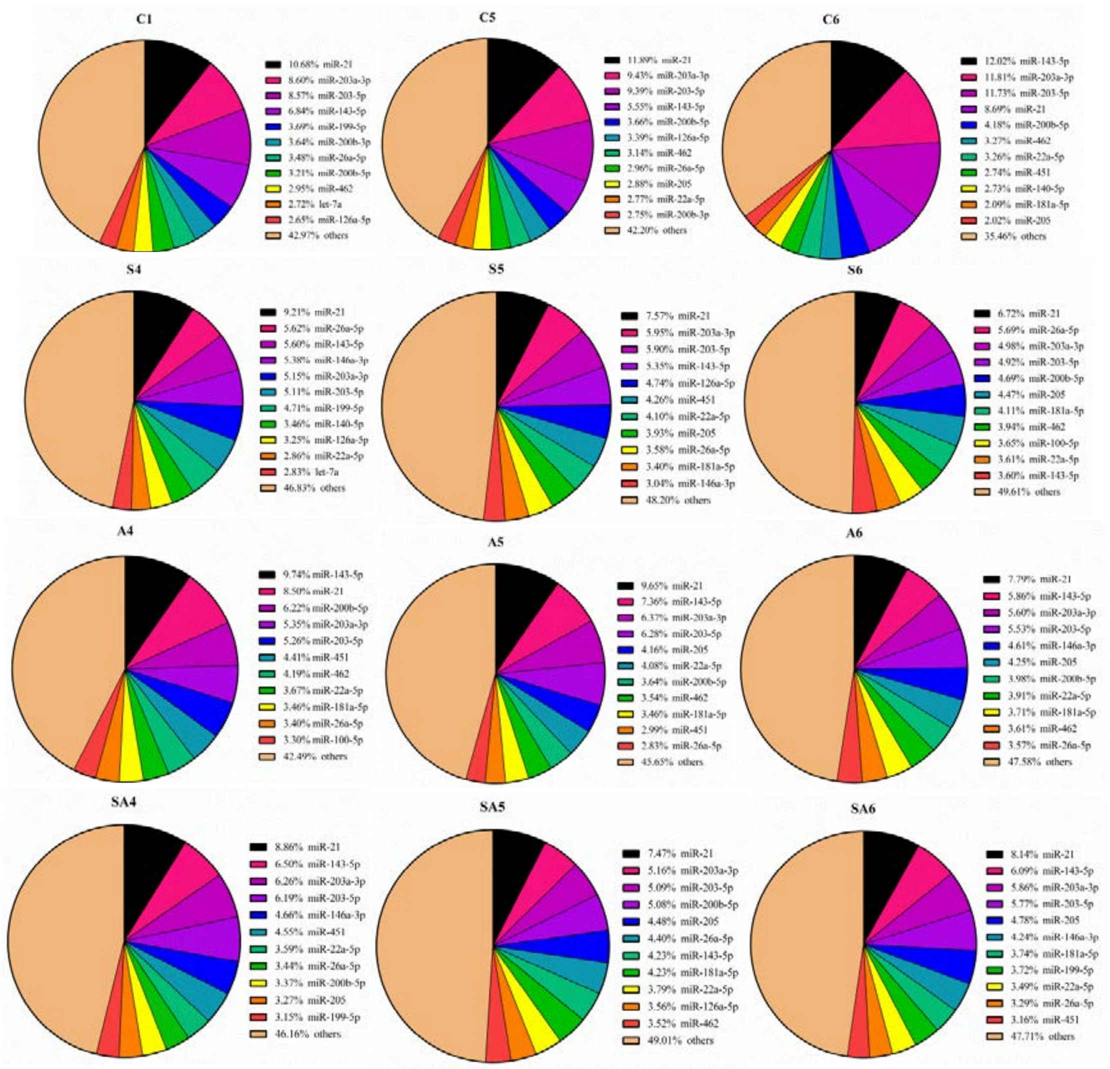

Suppl. FIGURE 2

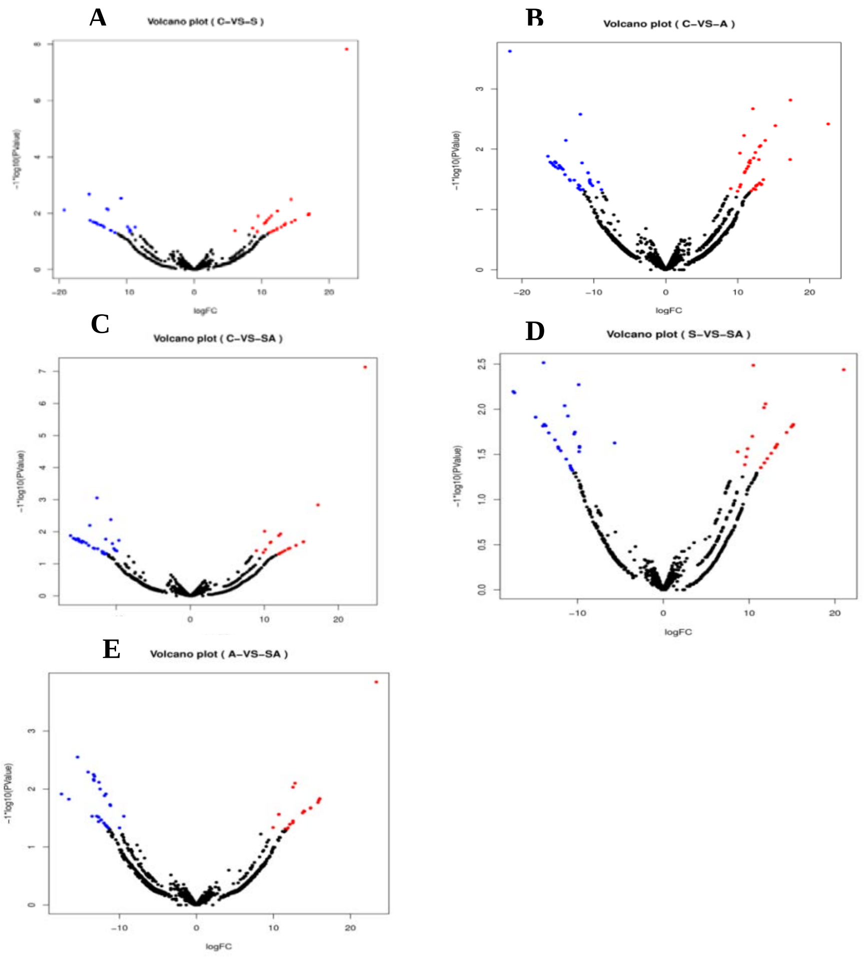

Suppl. FIGURE 3

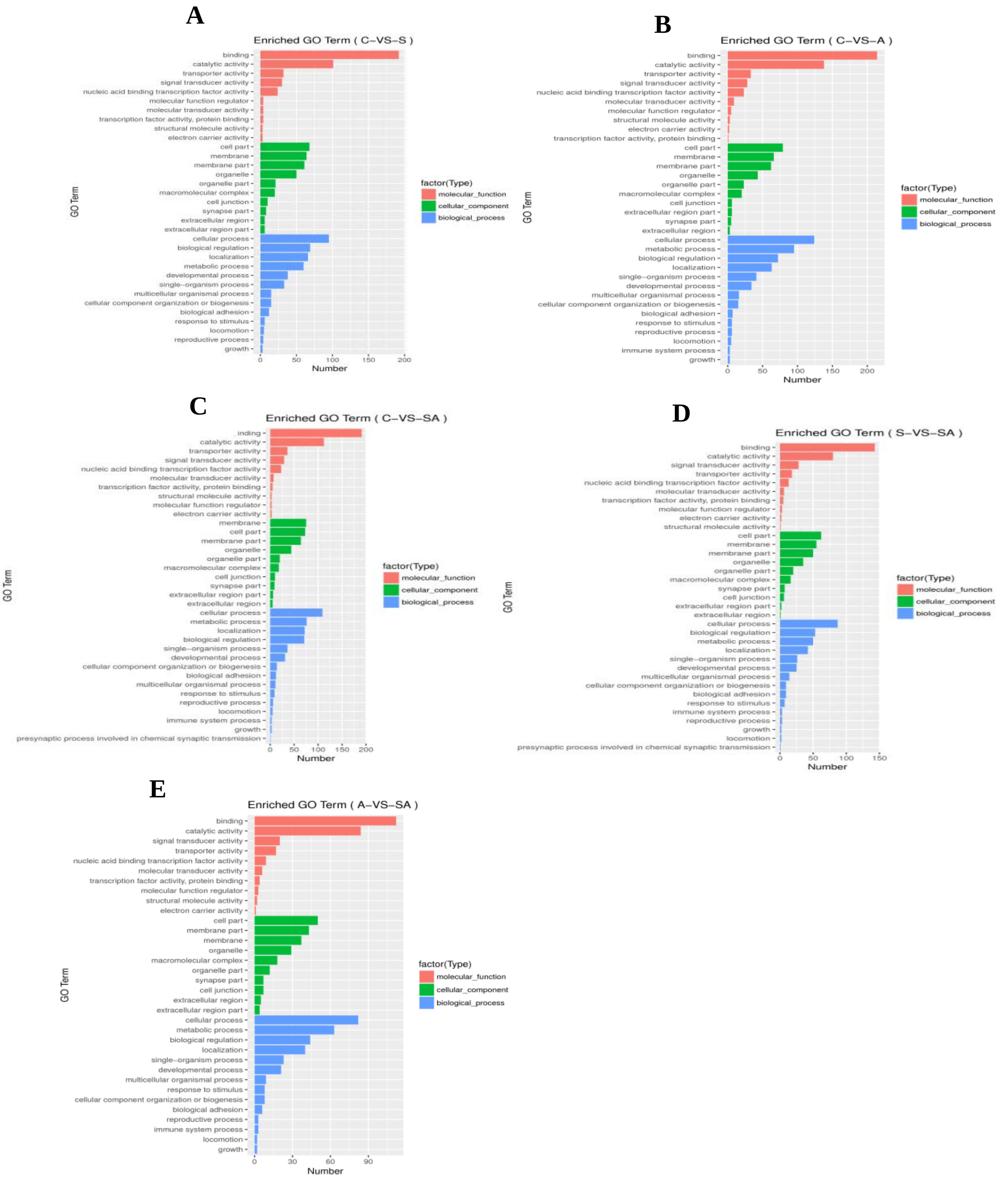

Suppl. FIGURE 4

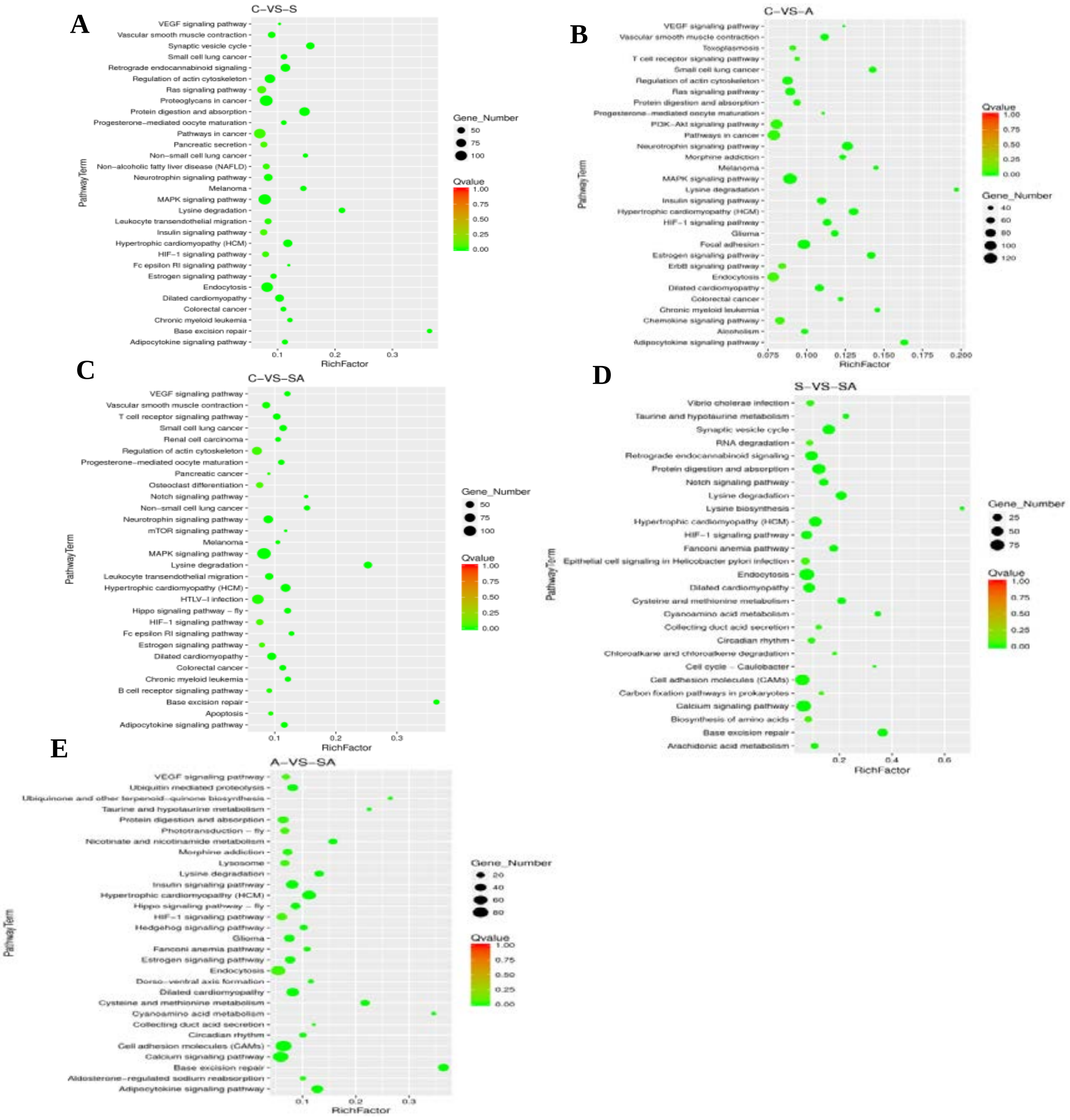

Suppl. FIGURE 5
